# Ultrasensitive label-free miRNA-21 detection based on MXene-enhanced plasmonic lateral displacement measurement

**DOI:** 10.1515/nanoph-2023-0432

**Published:** 2023-10-11

**Authors:** Yuye Wang, Yurui Hu, Ruibin Xie, Qi Zeng, Yanhang Hong, Xi Chen, Pengcheng Zhang, Lin Zeng, Yi Zhang, Shuwen Zeng, Hui Yang

**Affiliations:** Research Center for Bionic Sensing and Intelligence, Institute of Biomedical and Health Engineering, Shenzhen Institute of Advanced Technology, Chinese Academy of Sciences, Shenzhen 518055, China; Light, Nanomaterials & Nanotechnologies (L2n), CNRS-EMR 7004, Université de Technologie de Troyes, 10000 Troyes, France

**Keywords:** miRNA detection, surface plasmon resonance, MXene-enhanced biosensing, phase singularity

## Abstract

miRNAs are small non-coding RNA molecules which serve as promising biomarkers due to their important roles in the development and progression of various cancer types. The detection of miRNAs is of vital importance to the early-stage diagnostics and prognostics of multiple diseases. However, traditional detection strategies have faced some challenges owing to the intrinsic characteristics of miRNAs including small size, short sequence length, low concentration level and high sequence homology in complex real samples. To overcome these challenges, we proposed a MXene-enhanced plasmonic biosensor for real-time and label-free detection of miRNA. By utilizing MXene nanomaterial which possesses unique characteristics including large surface area and strong carrier confinement abilities, we tuned the absorption of our plasmonic sensing substrate to reach a “zero-reflection” state and induced an extremely sharp phase change at the resonance angle. Combined with the sensing mechanism based on phase-induced lateral displacement measurement, this MXene-enhanced plasmonic biosensor can achieve a much superior sensing performance compared to traditional SPR devices. Based on this biosensing scheme, the ultrasensitive detection of target miRNA with a detection limit down to 10 fM has been successfully demonstrated. More importantly, single-base mismatched miRNA can be easily distinguished from the target miRNA according to the sensing signal. Furthermore, our plasmonic biosensor is capable of detecting miRNA in complex media such as 100 % human serum samples without compromising the detection sensitivity. This MXene-enhanced plasmonic sensing scheme has the ability of detecting miRNAs with extremely low concentration levels in complex surrounding media without the need of introducing extra labels or amplification tags, which holds great potential in various biological applications and clinical diagnostics.

## 1 Introduction

The abnormal expression of specific microRNAs (miRNAs) is closely related to different kinds of cancer [1–3]. Among them, miRNA-21 is a well-recognized biomarker that has been proven to be upregulated in many cancer types including breast, pancreatic, lung and colorectal cancers, etc. [4, 5]. As a result, the ultrasensitive detection of miRNA-21 is of vital significance to early-stage cancer diagnostics. However, the small size and low concentration level of miRNA-21 in clinical samples have made its detection quite challenging [6–8].

Traditional analytical strategies for miRNA detection include quantitative reverse transcription polymerase chain reaction (qRT-PCR) [9], microarray-based hybridization [10] and next-generation sequencing [11], etc. [12–14]. The qRT-PCR method has high sensitivity and specificity. However, its performance largely relies on the designed primers and sample preparation conditions [15]. Moreover, a reference gene is usually needed for signal normalization. Microarray-based hybridization shows unique advantages in multiplexed detection, but it fails to compete in terms of sensitivity. Also, high background noise may occur owing to non-specific hybridization [16]. Next-generation sequencing is capable of profiling both known and novel miRNAs. Nevertheless, sequencing bias and errors may be introduced in the detection process [17]. The high cost and computationally intensive operations also hinder its accessibility. Most importantly, all the above approaches require additional amplification or labeling procedures, which greatly limits the application scenarios.

Surface plasmon resonance (SPR) technology, which possesses the advantages of label-free, real-time, dynamic monitoring abilities and good stability, has become one of the most promising sensing strategies for miRNA detection [18]. Nevertheless, limited by the detection sensitivity, traditional SPR biosensors based on wavelength or angle are difficult to detect miRNAs with low concentration levels. It is also challenging to distinguish miRNAs with high sequence homology. To overcome these challenges, researchers have proposed various amplification strategies including exploiting gold nanoparticles as extra signal amplification tags [19, 20] and designing DNA tetrahedron structures [21, 22]. However, these strategies will add additional detection steps and complexity in time will be largely increased.

In this paper, we have proposed a MXene-enhanced plasmonic biosensor based on the detection of lateral displacement known as Goos–Hänchen (GH) shift for ultrasensitive miRNA detection. Over the recent years, MXene has been explored as a promising biosensing material due to its merits of layered structure, large surface area, strong carrier confinement abilities and high binding energies for biomolecules, etc. [23–25]. Here, we have designed an optimized MXene-on-Au substrate and combined it with the GH shift interrogation method to achieve optimized sensing performance for the first time. The designed plasmonic substrate was calibrated through reflectivity measurement, which is in good accordance with our simulation model. The capability of detecting minute refractive index (RI) changes was also demonstrated by measuring PBS solutions with extremely low concentration differences. Compared with traditional wavelength-based measurements, our device can reach a much higher sensitivity.

The sensing scheme was then applied to the detection of miRNA-21 with a detection limit down to 10 fM (10^−14^ mol/L). Moreover, single mismatched miRNA can be distinguished from the target miRNA-21, demonstrating the high specificity of our device and the ability to recognize miRNA sequences with high homology. More importantly, our plasmonic sensing scheme is capable of direct miRNA detection in complex media such as human serum, which broadens its application scenarios. We anticipate that our device will provide a useful tool in early-stage clinical diagnostics of various major diseases.

## 2 Materials and methods

### 2.1 Reagents

DNA and RNA oligonucleotides were synthesized by Sangon Biotechnology Co. (Shanghai, China). The DNA and miRNA sequences used in this work are listed here:

miRNA-21: 5′-UAGCUUAUCAGACUGAUGUUGA-3′

amine-modified DNA sequence: 5′-NH_2_-TTTTTCAACATCAGTCTGATAAGCTA-3′ (complementary to miRNA-21)

single base mismatch miRNA: 5′-UAGCUUAUCAUACUGAUGUUGA-3′

All DNA and RNA samples used in this work were dissolved in DNase/RNase-Free distilled water purchased from Fisher Scientific (USA). (3-Glycidyloxypropyl)trimethoxysilane (GOPTS), HCl, and NaOH were purchased from Sigma-Aldrich (USA).

### 2.2 Design of plasmonic sensing system

**Figure 1:**
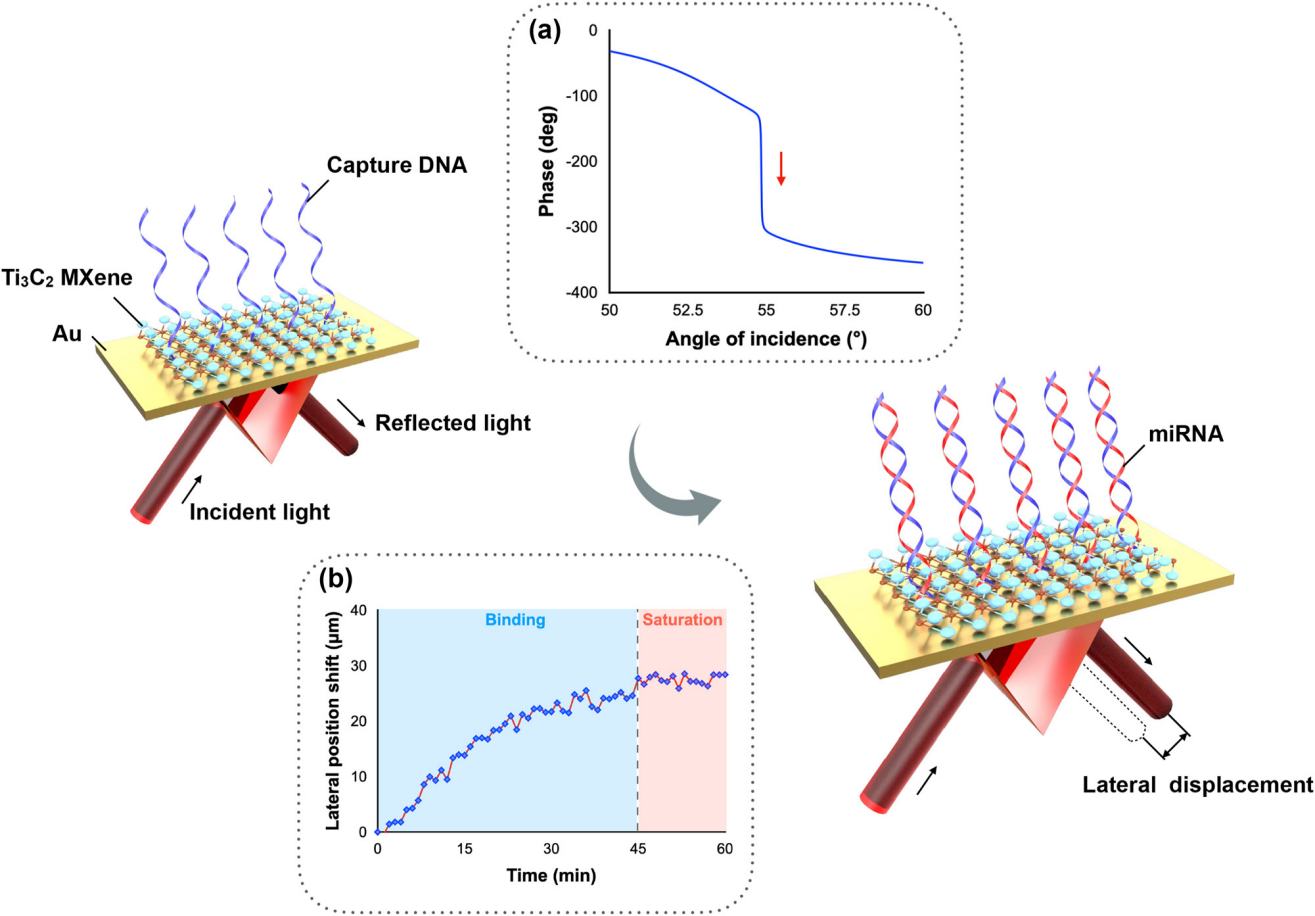
Schematic diagram of miRNA detection scheme based on phase-induced lateral displacement measurement. Inserted figure (a) Sensing mechanism based on a sharp phase change. (b) Sensing signal upon miRNA injection.

In our work, a plasmonic sensing system based on lateral displacement measurement is designed. Compared to traditional wavelength- or angle-based measurement, this scheme utilizes a phase-induced lateral position shift measurement that can achieve higher detection sensitivity. The schematic diagram of this biosensing system is shown in Figure 1. The plasmonic sensing substrate is functionalized with capture DNA first. When the target miRNA molecules are presented in the solutions, the reflected light beam experiences a sharp phase change and a large lateral displacement is readily detected by a position sensor. By dynamically monitoring the change in the position shift, real-time and direct detection of miRNA can be realized. This lateral displacement is also known as the GH shift, which is defined as the higher order of phase signal. According to the basic principle of SPR, when the incident light reaches the plasmonic substrate at a specific angle known as the SPR angle, a propagating surface plasmon is excited, generating a significant decrease in the intensity of the reflected beam. This state is known as “zero-reflection”, during which a sharp phase change also occurs. Compared to the change in intensity or SPR angle, the change in optical phase is much steeper and therefore leads to much higher sensitivity. The sharp phase change results in a giant lateral displacement [26–28]. The information on lateral displacement change can be easily extracted from a commercial position sensor, which largely decreases the detection complexity without compromising the high level of sensitivity [29]. The schematic illustration of this GH shift-based SPR optical setup is presented in Supporting information.

The improvement in detection sensitivity largely depends on the optimization of the plasmonic sensing substrate to reach a perfect absorption state. When the reflection reaches almost zero, the sharpest optical phase change and the largest lateral displacement change take place, meaning that the highest possible detection sensitivity can be realized when the lowest possible reflection is achieved at the SPR angle. In conventional SPR substrates that consist of noble metals only (e.g., gold, silver, copper, etc.) [30, 31], a strict requirement on controlling the metal thickness should be obeyed to optimize the detection sensitivity [32]. However, current fabrication technologies cannot meet the high demand for tuning the thickness to the resolution of a few nanometers. To overcome this challenge, we have utilized MXene as an effective tuning material. Through adding a thin layer of MXene nanosheets, the reflection can be significantly reduced, which in turn leads to a much sharper optical phase signal change and the corresponding lateral position shift change.

To optimize the sensing substrate, a detailed simulation analysis was performed. In a multilayer Kretchmann configuration with *n* layers, the reflectivity can be calculated as:

(1) $$\begin{array}{c} R=r_{1,n}=\frac{r_{1,\text{n}-1}+r_{n-1,n}\text{exp}\left(2\text{i}k_{n-1}d_{n-1}\right)}{1+r_{1,\text{n}-1}r_{n-1,n}\text{exp}\left(2\text{i}k_{n-1}d_{n-1}\right)} \end{array}$$

Therefore, to calculate the reflectivity, we need to sequentially identify *r*_1,2_, *r*_1,3_ … to *r*_1,*n*−1_.

The reflection coefficient *r*_*n*−1,*n*_ of two adjacent layers (*n*th layer and (*n* − 1)th layer) is

(2) $$\begin{array}{c} r_{n-1,n}=\frac{Z_{n-1}-Z_{n}}{Z_{n-1}+Z_{n}} \end{array}$$

where $Z_{n}=\frac{\varepsilon_{n}}{k_{n}}$ , which is the ratio between the complex dielectric constants of the *n*th layer (*ɛ*_*n*_) and the wave vector in the *n*th layer (*k*_*n*_). *k*_*n*_ can be calculated by $k_{n}=k_{0}\sqrt{\varepsilon_{n}-\varepsilon_{n}\sin^{2}\theta_{c}}$ . In these equations, *k*_0_ is the wave vector of light in free space, *θ*_*c*_ is the incident angle, *d*_*n*_ is the thickness of the *n*th layer.

The complex reflection coefficients can be represented as *r* = |*r*|exp(i*ϕ*), where *ϕ* denotes the optical phase of the light. Here, we measured the lateral displacement signal, which is more easily accessible than the phase signal. This displacement is known as the higher-order mode of the phase signal and can be represented by [33]:

(3) $$\begin{array}{c} \Delta L=-\frac{1}{k_{0}}\frac{\partial \phi }{\partial \theta } \end{array}$$

**Figure 2:**
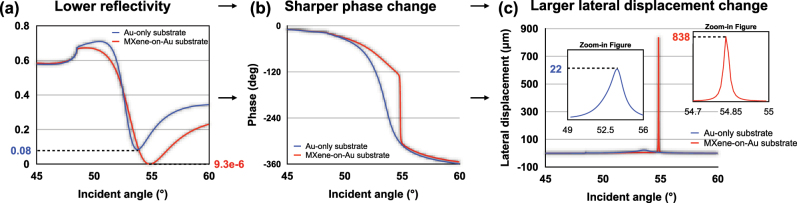
Simulation analysis of (a) reflectivity (b) phase (c) lateral displacement signal based on both Au-only substrate and MXene-enhanced substrate.

According to the above equations, we have simulated the reflection state of the MXene-enhanced substrate by a MATLAB program. The detailed optimization process and the parameters used in the simulation have been listed in the Supporting information. In Figure 2(a), we can see that the minimum reflectivity can be reduced from 0.08 to 9.3 × 10^−6^ by changing the traditional sensing substrate to the MXene-on-Au configuration. As discussed earlier, when the minimum reflectivity is greatly reduced, a much sharper phase change is obtained (Figure 2(b)) and a larger lateral displacement change is achieved. As shown in Figure 2(c), the largest lateral displacement can be increased from 22 μm to up to 838 μm, demonstrating that a more significant signal change can be detected upon molecular binding at the sensing interface.

### 2.3 Substrate fabrication and surface functionalization

The plasmonic substrate was designed and fabricated with the optimized parameters based on the simulation analysis, as introduced in the last section. First, 40 nm Au, as well as 2.5 nm Ti as the adhesion layer, were fabricated onto the BK7 glass substrate through DC magnetron sputtering. Then, we spin-coated Ti_3_C_2_ MXene (single layer colloidal solution) with a concentration of 0.8 mg/mL as a tunable layer onto the Au-coated substrate. The spin-coating parameters used in our experiments are 3000 rpm with a duration of 10 s. Compared to the fabrication of a traditional Au-only SPR substrate, only a quick spin-coating procedure is needed to fabricate our optimized MXene-enhanced plasmonic substrate, which barely adds any complexity to the fabrication process.

To detect target miRNA with specific sequences, capture DNA was designed and functionalized onto the MXene-on-Au substrate using GOPTS as the linker. The plasmonic substrate was immersed in 2 % GOPTS (dissolved in ethanol) for 1 h to enable the covalent bonding between the epoxide group of GOPTS and the functional groups of MXene [34]. Then, the chamber was filled with ultrapure water to wash away the unbound GOPTS. After that, the synthesized NH_2_-functionalized DNA was injected into the reaction chamber and placed for 1 h 20 min. The plasmonic substrate was therefore functionalized with capture DNA with a sequence complementary to miRNA-21. To avoid nonspecific binding, 1 % BSA solution which is commonly used as the blocking agent [35, 36], was introduced to the surface for 40 min. After washing with distilled water, the substrate was ready for the following direct miRNA detection. Finally, the plasmonic substrate was regenerated using 2.5 mM HCl solutions and 10 mM NaOH/0.1 % SDS solutions.

## 3 Result and discussion

### 3.1 System calibration

**Figure 3:**
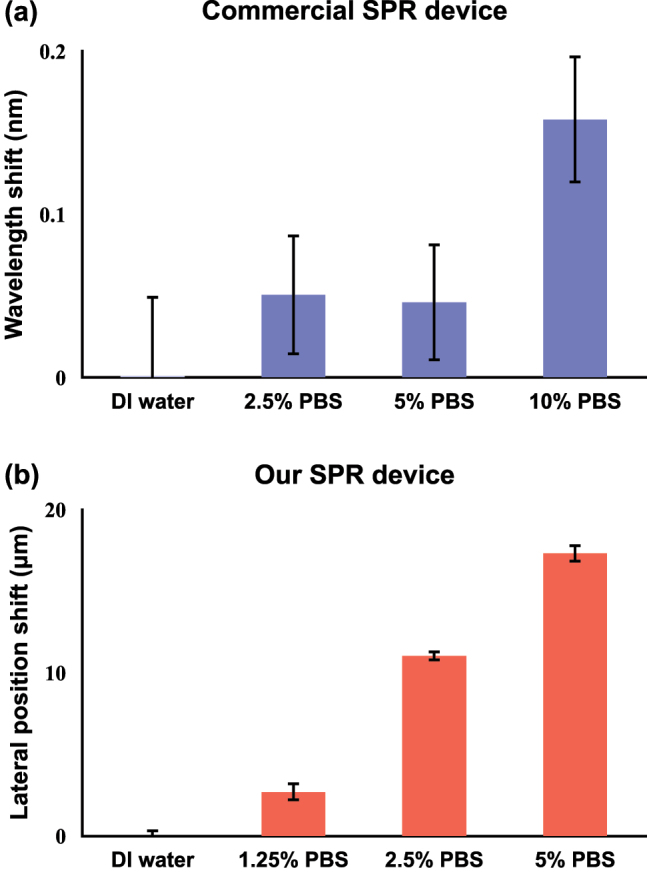
Calibration results of measuring PBS with different concentration levels using (a) a commercial SPR device based on wavelength shift and (b) our SPR device based on lateral position shift.

First, we have demonstrated the superiority of our plasmonic biosensing platform based on lateral position shifts over the traditional wavelength interrogation method. As explained earlier, the GH shift-based measurement has the capability of achieving a sharper signal change compared to wavelength or angle-based detection. Here, we tested the performance of a commercial SPR device (P4SPR, Affinité Instruments) that utilizes the typical wavelength interrogation method. As shown in Figure 3(a), the wavelength shift signal of 2.5 % (defined as the volume ratio between 1 × PBS and distilled water) PBS solutions and 5 % PBS solutions showed no significant difference. The results indicated that the commercial device failed to distinguish small refractive index variations. In Figure 3(b), we have demonstrated the performance of our device in detecting minute RI changes. Unlike Figure 3(a), there is a significant difference in lateral position signal change between 2.5 % and 5 % PBS, which proved that our device possessed higher sensitivity. Moreover, our device can distinguish even lower PBS concentration (1.25 %), which corresponds to a lower detection limit compared to the commercial one. It is also noticeable that the standard deviations of data acquired by our device were much smaller than the commercial one, which revealed the higher stability and signal-to-noise ratio of our device.

**Figure 4:**
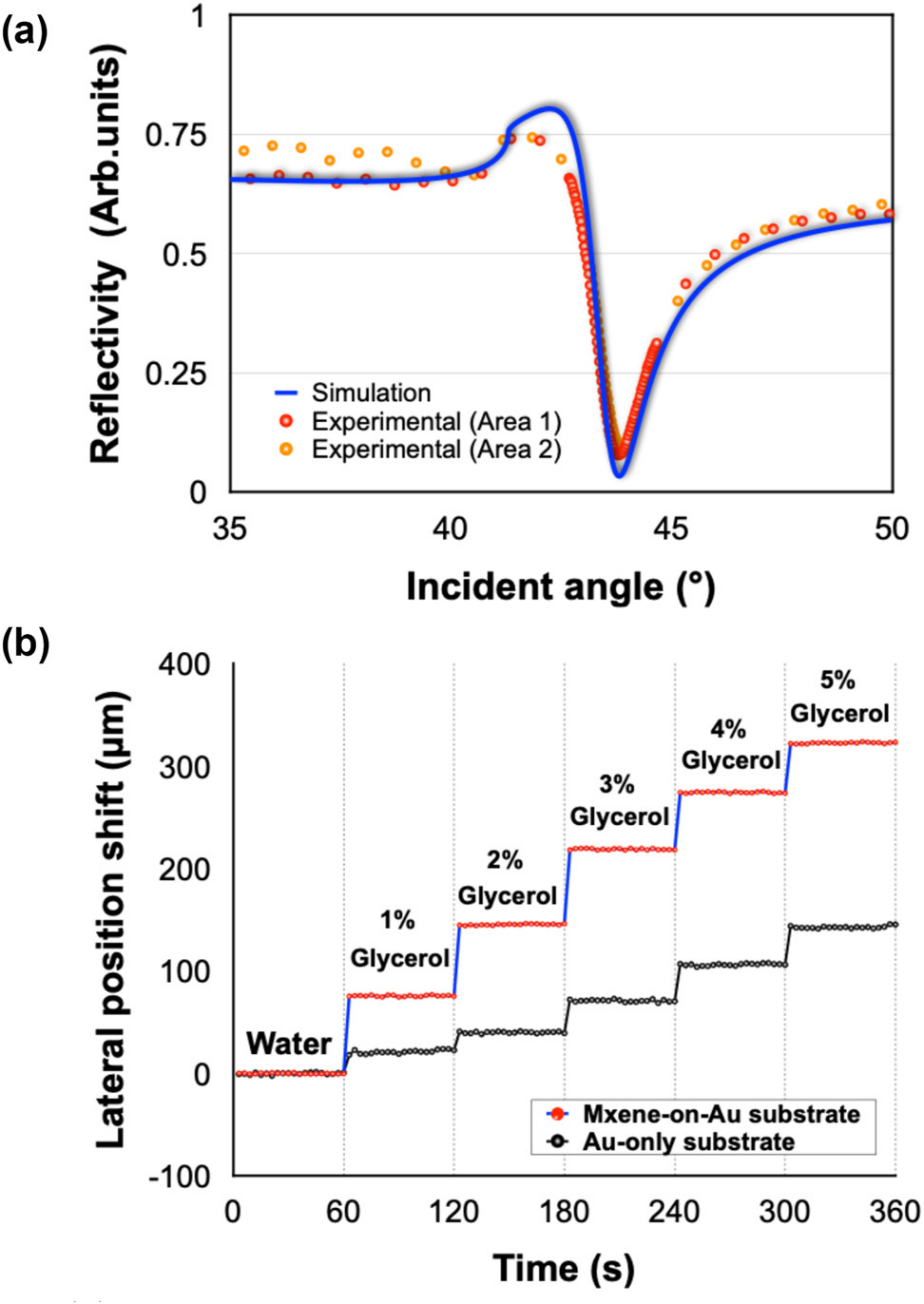
Calibration tests on the designed plasmonic substrates: (a) Simulation and experimental results of reflectivity spectra in air based on MXene-on-Au substrate. (b) Lateral position shift signal of glycerol solutions with different concentration levels based on MXene-on-Au and Au-only substrate.

To further enhance the detection sensitivity, we have utilized MXene nanosheets to tune the absorption of our plasmonic sensing substrate. To validate the reliability of our device, we measured the reflectivity of the MXene-on-Au substrate in air and compared the experimental results with our simulation analysis. We randomly selected two points on the substrate and plotted the results in Figure 4(a). Two sets of experimental data showed no significant variations with each other and were both in good accordance with our simulation results, demonstrating the stability of our device and the capability of achieving comparable results with simulation calculations. Next, as a standard calibration test, we measured the change of lateral position shift signal with respect to glycerol solutions with different concentrations. As depicted in Figure 4(b), a much higher signal response upon injection and larger signal change between different concentration levels was achieved using our optimized MXene-on-Au substrate compared with the traditional Au-only substrate. The bulk sensitivity of our device can reach the level of 10^4^ μm/RIU. The calibration results indicated that higher sensing performance can be readily realized by utilizing a layer of MXene material.

### 3.2 Direct miRNA detection

**Figure 5:**
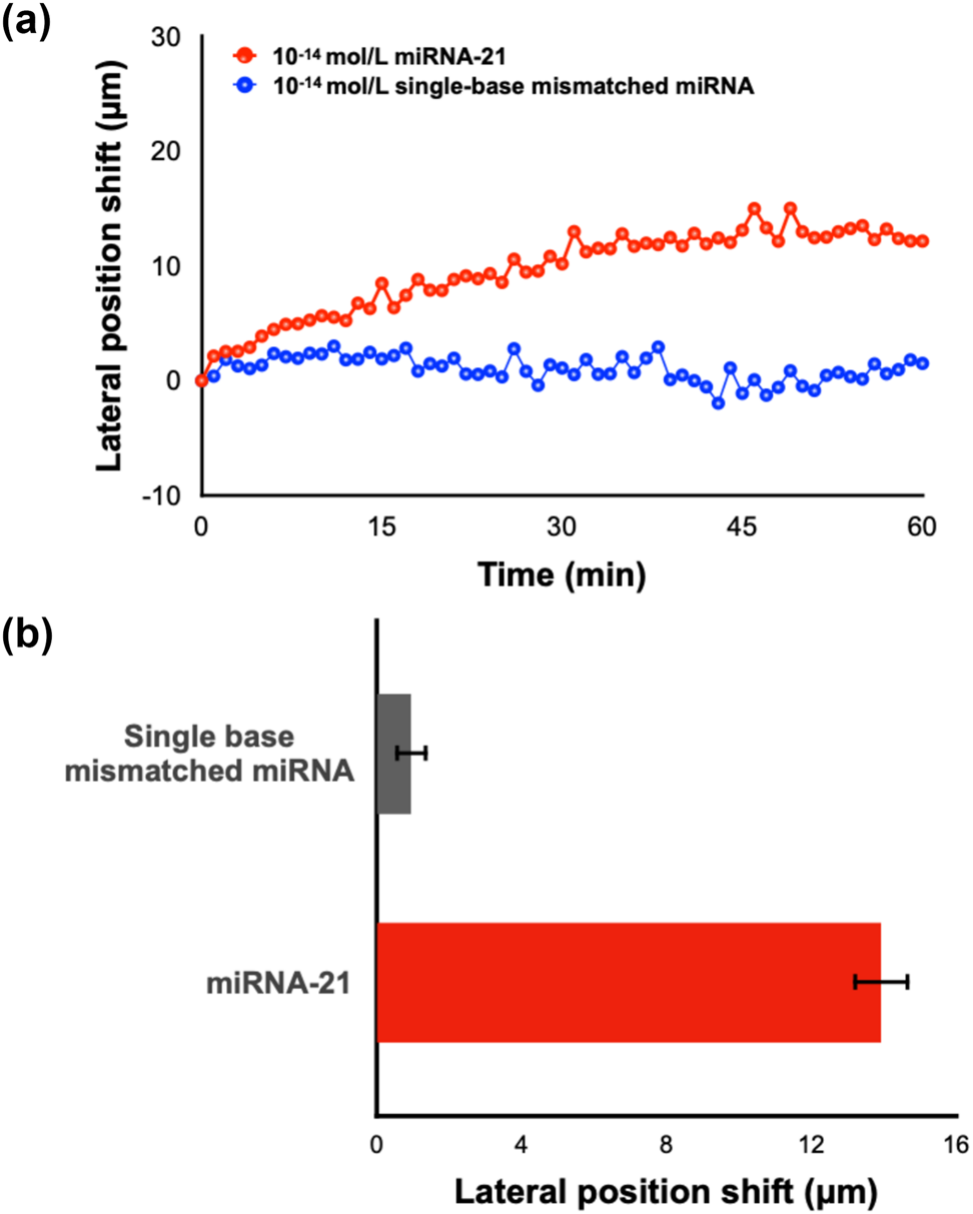
Direct detection of miRNA-21. (a) Real-time lateral position shift measurement of miRNA-21 and single base mismatched miRNA. (b) Result summary of repeated experiments.

The capability of this MXene-enhanced plasmonic system in ultrasensitive biosensing was demonstrated by the detection of miRNA-21. As shown in Figure 5(a), the lateral position shift signal gradually increased upon the injection of target molecules and saturated after 20–30 min. This trend in the measured curve indicated the efficient binding between miRNA-21 and its complementary capture DNA on the substrate. The lowest detectable concentration of miRNA-21 was obtained at 10 fM, which is enhanced by two orders of magnitude compared to the commercial SPR device (details in Figure S6, Supporting information). Subsequently, to test the specificity of this device, single base mismatched miRNA with the same concentration level as miRNA-21 was introduced into the reaction chamber as well. No observable signal change was detected during the whole process. The two entirely different binding curves in Figure 5(a) indicated that only perfectly matched miRNA can cause a large lateral position shift signal, which demonstrated the high specificity of our device.

Both experiments (detection of miRNA-21 and single base mismatched miRNA) were repeated three times. A summary of these multiple sets of experiments including the average lateral displacement signal value and standard deviations is shown in Figure 5(b). We can see that the single base mismatched miRNA barely induced any signal change in the system while the miRNA-21 can cause an average signal increase of 13.93 μm. The dataset also presented a high consistency between different sets of experiments, which proved the stability and reliability of our device. Moreover, the six sets of experiments were conducted using the same MXene-on-Au substrate. Each detection run was carried out after surface regeneration and surface functionalization procedures, which proved the reproducibility of our plasmonic sensing substrate. We have also tested the ability of our system in detecting miRNA-21 with various concentrations. Our investigations have substantiated that the proposed system has a measurement range of miRNA detection from 10 fM to 10 nM. The lateral displacement signal has increased when increasing the concentration of miRNA-21. Notably, this increment demonstrated a trend of reaching a saturation point beyond a concentration level of 10^−10^ mol/L, as illustrated in Figures S7 and S8 (Supporting information).

### 3.3 miRNA detection in human serum samples

**Figure 6:**
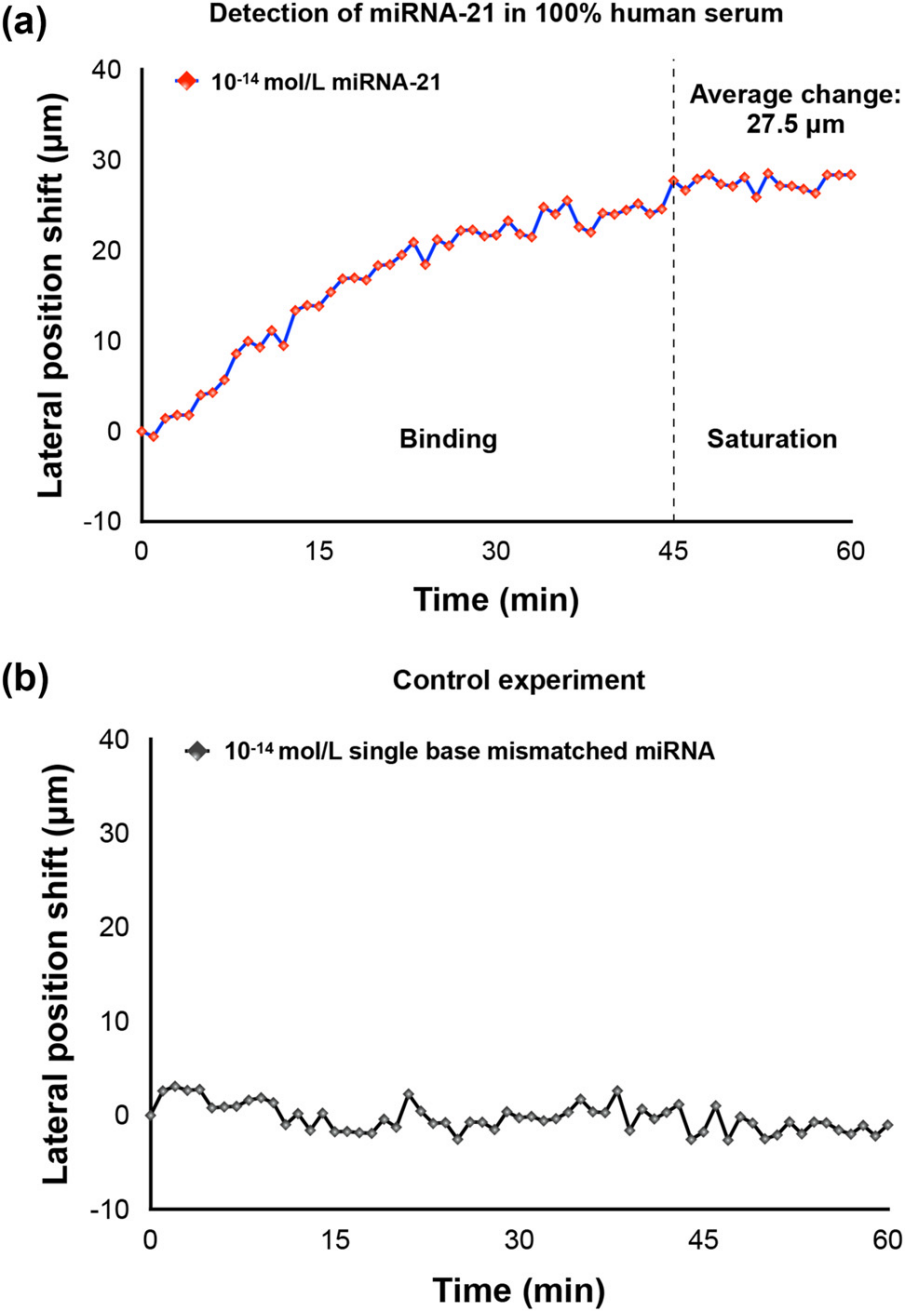
Detection of miRNA in 100 % human serum samples. (a) Detection of miRNA-21. (b) Detection of single base mismatched miRNA.

To demonstrate the capability of our device in detecting miRNAs in real clinical samples, we have tested it with miRNA-21 spiked into an undiluted human serum sample. As shown in Figure 6, the trend of the sensing signal is similar despite the change of detection media from PBS solutions to 100 % human serum. In Figure 6(a), the lateral displacement also experienced a gradual binding process and saturated at around 45 min. The average lateral position shift change after saturation is measured to be around 27.5 μm. Only miRNA-21 with sequence specific to the capture DNA caused a gradually increased lateral displacement signal while single base mismatched miRNA barely induced any change to the sensing signal, as shown in Figure 6(b). The detection limit of miRNA in human serum can also reach 10^−14^ mol/L. The experimental results have verified that the abundance of protein in the human serum did not compromise the sensing performance of our plasmonic biosensor.

## 4 Conclusions

In this paper, we have proposed an ultrasensitive plasmonic sensing scheme for label-free and real-time miRNA detection based on MXene-enhanced substrate. Combined with GH-shift measurement which possesses high sensitivity, miRNA-21 with concentration down to 10 fM has been successfully detected. Single-base mismatched miRNA has been effectively recognized from the sensing signal and binding curve, which demonstrates the high specificity of our device. More importantly, the detection of low-concentration miRNA in complex media such as 100 % human serum has also been achieved. This MXene-enhanced SPR biosensor based on the lateral displacement detection mechanism offers superior sensing performance to traditional SPR techniques and is capable of direct detection without the need to introduce extra labels or amplification tags. Our biosensing technique that effectively exploits the unique properties of MXene to enhance SPR performance provides a promising tool for the effective detection of miRNA and could evolve into a platform to detect a wide class of nano-objects, such as many other tumor biomarkers in clinical diagnosis, viral particles, or for monitoring synthesis and aggregation processes on a molecular level.

## Supplementary Material

Supplementary Material Details
