# Supplementary material for: Ultrasensitive label-free miRNA-21 detection based on MXene-enhanced plasmonic lateral displacement measurement
Source: Nanophotonics. 2023 Oct 11;12(21):4055–62. doi: 10.1515/nanoph-2023-0432 (PMC11501375; doi:10.1515/nanoph-2023-0432)
Supplement: Supplementary file 1 — Supplementary Material Details [file j_nanoph-2023-0432_suppl_001.pdf]

Supplementary Information

Ultrasensitive label-free miRNA-21 detection based on MXene-enhanced plasmonic lateral displacement measurement

Yuye Wang[1], Yurui Hu[1], Ruibin Xie[1], Qi Zeng[1], Yanhang Hong[1], Xi Chen[1], Pengcheng Zhang[1], Lin Zeng[1], Yi Zhang[1], Shuwen Zeng[2], Hui Yang[1*]

[1] *Institute of Biomedical and Health Engineering, Shenzhen Institute of Advanced Technology, Chinese Academy of Sciences, Shenzhen, China.*

[2] *Light, Nanomaterials & Nanotechnologies (L2n), CNRS-EMR 7004, Université de Technologie de Troyes, 10000 Troyes, France.*

Optimization of the plasmonic sensing substrate

Table S1 Parameters used in optimizing the sensing substrate

Layer	Material	Refractive index (Incident wavelength =633 nm)
I	**Prism SF11**	1.7786
II	**Ti**	2.7039+3.7652 i
III	**Au**	0.191+3.6656 i
IV	**MXene**	2.88+1.33 i [1,2]
V	**Sensing medium**	1.3317

The reflection is tuned by optimizing the thickness of each layer in the Kretschmann configuration. The parameters used to optimize the sensing substrate are shown in Table S1. The thickness of MXene was tuned from 0 nm to 6 nm. As shown in Figure S1, the total reflectivity of the sensing substrate will first decrease and then experience an increase.

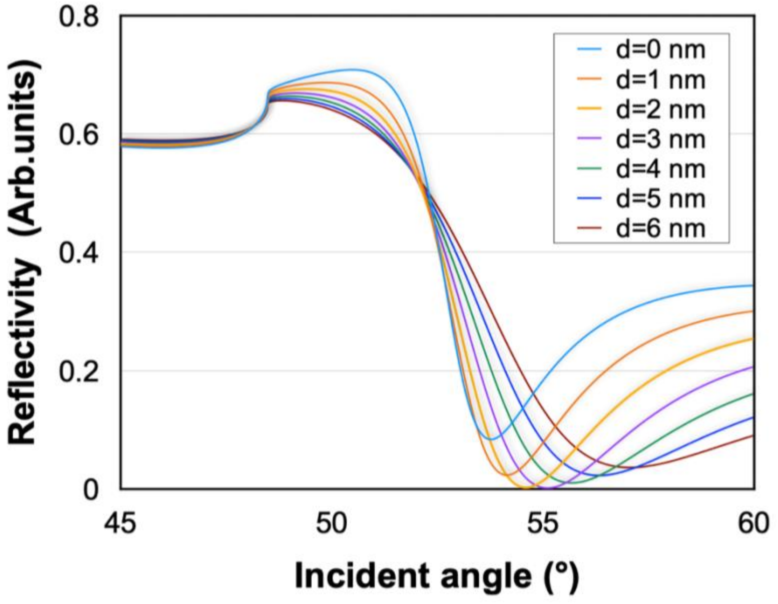

Figure S1 Optimization of reflectivity by tuning the thickness of MXene.

Also, when the thickness of Au changes from 35 to 50 nm, the reflection will also be affected. Therefore, we need to tune the parameters to achieve the "almost zero" state. The optimized parameters we used for the plasmonic sensing substrate are 2.5 nm Ti, 40 nm Au, and 2-3 nm MXene. The sensitivity of SPR sensing based on lateral displacement is in close relationship with the reflectivity. The bulk sensitivity is defined as $S = \frac{\Delta L}{\Delta n}$ (the change in lateral displacement with respect to the changes in refractive index) and can be quantified through the Matlab program. Here, we simulated the signal change when the refractive index of the sensing medium changed. In our optimized setting, minute refractive index changes (10^{-6} RIU) can also be detected with a bulk sensitivity of $2.96\times 10^{6}\ \mu m/RIU$. Figure S2 showed the bulk sensitivity of the MXene-on-Au substrate and Au-only substrate.

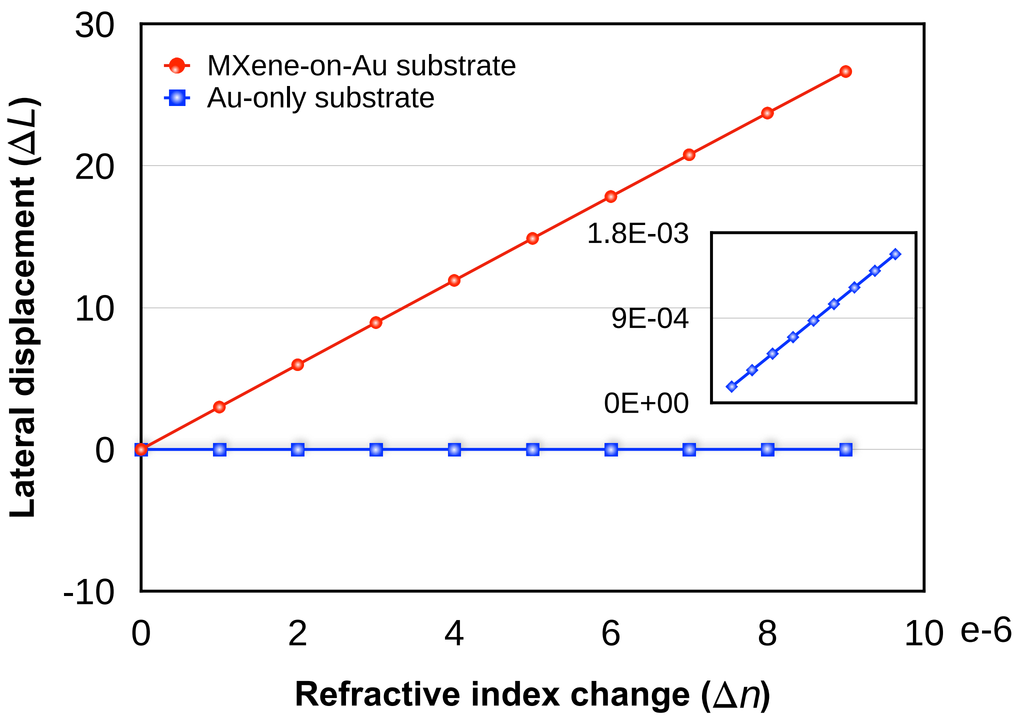

Figure S2 Lateral displacement changes with respect to the refractive index changes based on MXene-on-Au and Au-only substrate.

Experimental setup

The GH shift-based SPR plasmonic sensing system is depicted in Fig.S3. A commercial position sensor is employed to detect the lateral displacement associated with the molecule binding process. We have used a He-Ne laser (Wavelength: 632.8 nm) as the light source. As shown in the figure, the incident light beam is split into *p*-polarized and *s*-polarized lights by a beam splitter and pass through an optical chopper. The light beams will reach the sensing substrate through prism coupling. The prism is fixed at the SPR resonance angle for obtaining a large lateral position shift signal change. A microfluidic reaction chamber is designed here to be integrated with the plasmonic substrate, which ensures the convenient transportation of target analytes onto the sensing region. As shown in Figure S4, the reaction chamber is made of PMMA and placed on top of the multi-layered plasmonic substrate with an o-ring in between to seal the liquid inside the PMMA chamber. The differential signal between *p*-polarized and *s*-polarized light will be measured by a position sensor to achieve a high signal-to-noise ratio since only *p*-polarized

light will respond to the SPR effect. The results are collected and analyzed using LABVIEW and MATLAB programs.

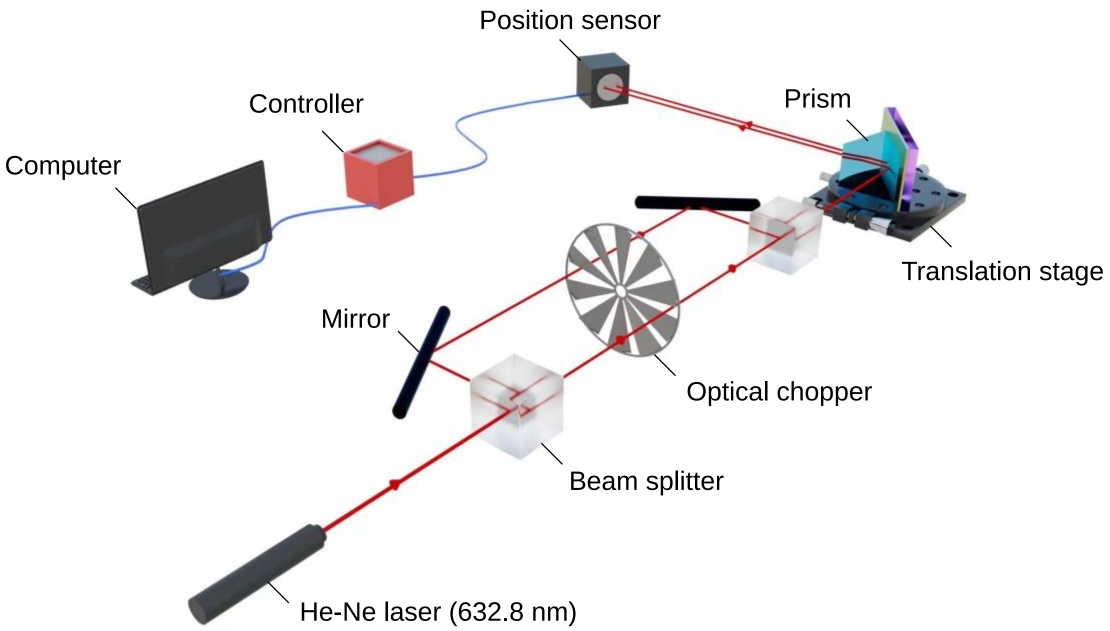

Figure S3 GH shift-based SPR optical setup.

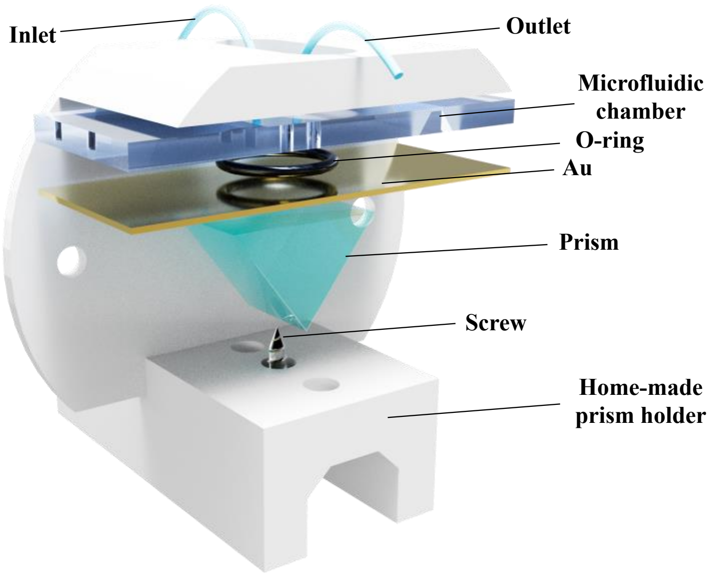

Figure S4 Detailed schematic diagram of the integrated SPR sensing module including prism, SPR substrate and the reaction chamber.

Characterization of MXene nanosheets

The monolayer colloid MXene solutions were purchased from Beike 2D materials Co.Ltd. We have characterized the morphology and thickness of MXene nanosheets through Atomic Force Microscopy (AFM). The thickness of monolayer MXene nanosheets is measured to be 0.89 nm on average.

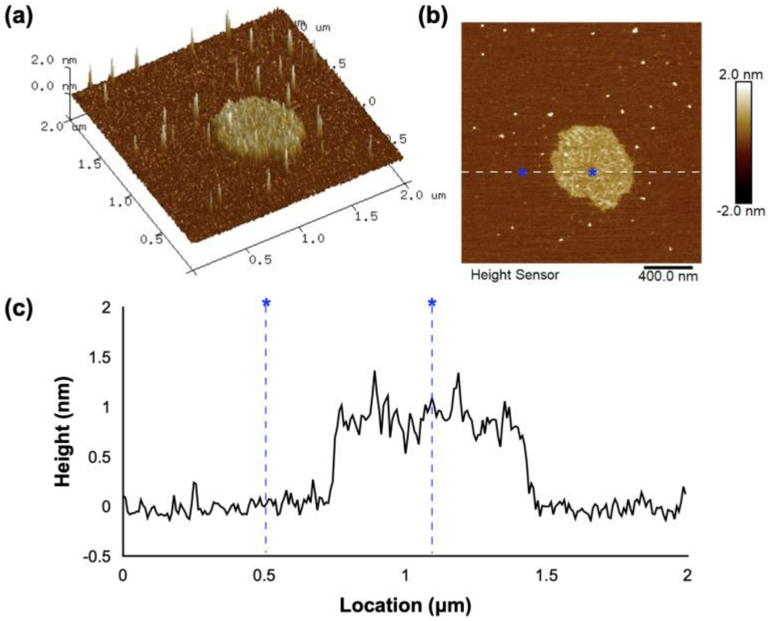

Figure S5 Characterization of MXene nanosheets.

Detection of miRNA-21 based on conventional SPR device

To further demonstrate the superiority of the proposed sensing method, we have done some sensing experiments based on the conventional SPR method. As shown in Figure S6, the wavelength shift is negligible when detecting miRNA-21 with a concentration level down to 10^{-13} mol/L. When the concentration increased to 1 pM, the wavelength shift signal turned out to be detectable. The total wavelength shift was detected to be around 0.26 nm. These results showed that the detection limit of the conventional SPR scheme is around 10^{-12} mol/L, which is two orders of magnitude higher than our proposed sensing scheme.

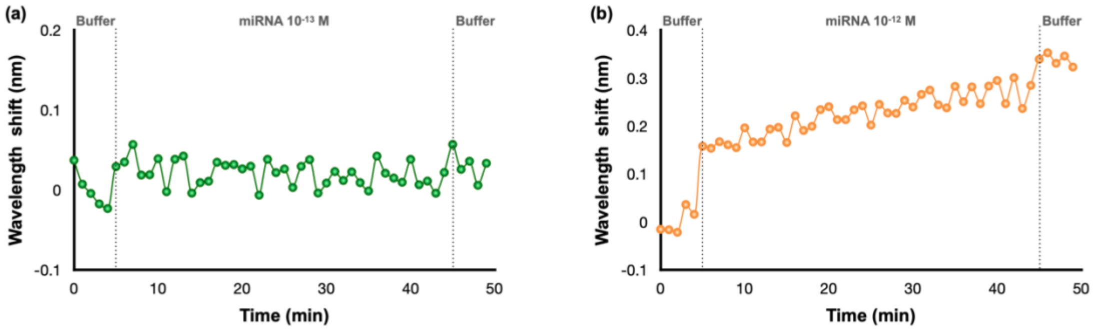

Figure S6 Detection of miRNA-21 based on commercial SPR device.

Detection of miRNA-21 with different concentration levels

miRNA-21 with various concentrations from 10 fM to 10 nM has been detected using our proposed SPR sensing scheme. The lateral displacement signal has increased when increasing the concentration of miRNA-21, as illustrated in Figure S7. Notably, this increment demonstrated a trend of reaching a saturation point beyond a concentration level of 10^{-10} M.

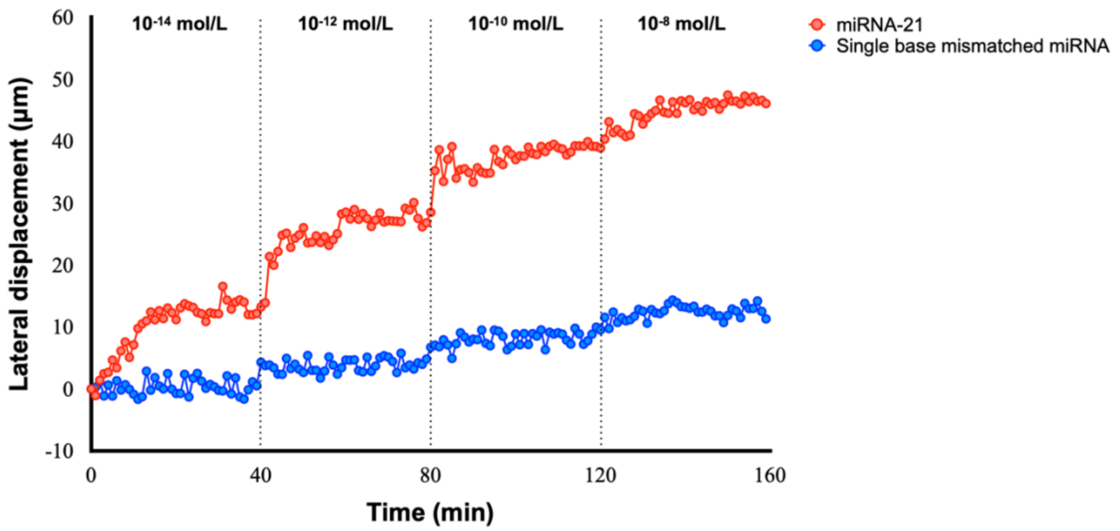

Figure S7 Real-time lateral displacement signal change in miRNA detection.

The detection experiments on miRNA-21 with various concentration levels have been repeated three times. Figure S8 presented a summary of these measurements, which showed the capability of distinguishing single base mismatched miRNA with a detection range from 10 fM to 10 nM.

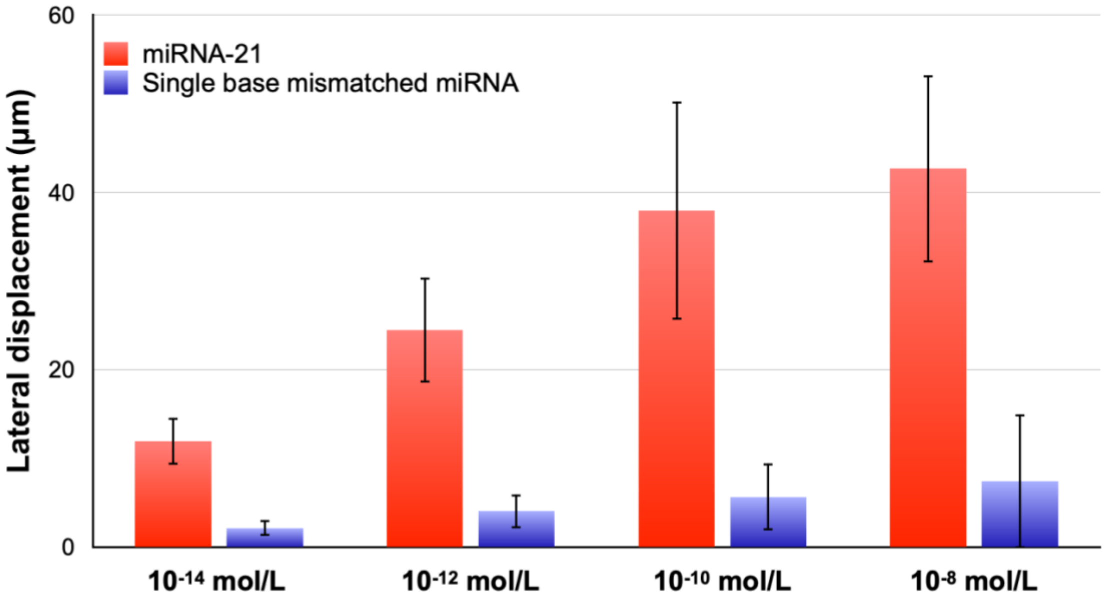

Figure S8 Summary of the detection results ranging from 10 fM to 10 nM.

Reference

[1] Wu, Leiming, et al. "Few-layer $Ti_3C_2T_x$ MXene: A promising surface plasmon resonance biosensing material to enhance the sensitivity." Sensors and Actuators B: Chemical 277 (2018): 210-215.

[2] Srivastava, Akash, et al. "A theoretical approach to improve the performance of SPR biosensor using MXene and black phosphorus." Optik 203 (2020): 163430.
